# Measurement Method of Stress in High-Voltage Cable Accessories Based on Ultrasonic Longitudinal Wave Attenuation

**DOI:** 10.3390/s24175843

**Published:** 2024-09-09

**Authors:** Jingang Su, Peng Zhang, Xingwang Huang, Xianhai Pang

**Affiliations:** State Grid Hebei Electric Power Co., Ltd., Electric Power Science Research Institute, Shijiazhuang 050022, China; hbdyyzp@163.com (P.Z.); huangxingwang1997@163.com (X.H.); yanerwon@163.com (X.P.)

**Keywords:** high-voltage cable accessories, cold shrink tube, stress, ultrasonic longitudinal wave, attenuation

## Abstract

High-voltage cables are the main arteries of urban power supply. Cable accessories are connecting components between different sections of cables or between cables and other electrical equipment. The stress in the cold shrink tube of cable accessories is a key parameter to ensure the stable operation of the power system. This paper attempts to explore a method for measuring the stress in the cold shrink tube of high-voltage cable accessories based on ultrasonic longitudinal wave attenuation. Firstly, a pulse ultrasonic longitudinal wave testing system based on FPGA is designed, where the ultrasonic sensor operates in a single-transmit, single-receive mode with a frequency of 3 MHz, a repetition frequency of 50 Hz, and a data acquisition and transmission frequency of 40 MHz. Then, through experiments and theoretical calculations, the transmission and attenuation characteristics of ultrasonic longitudinal waves in multi-layer elastic media are studied, revealing an exponential relationship between ultrasonic wave attenuation and the thickness of the cold shrink tube. Finally, by establishing a theoretical model of the radial stress of the cold shrink tube, using the thickness of the cold shrink tube as an intermediate variable, an effective measurement of the stress of the cold shrink tube was achieved.

## 1. Introduction

High-voltage cables are the main arteries of urban power supply. Cable accessories connect different sections of cables or cables to other electrical equipment. The stress (compression force) in the cold shrink tube of cable accessories is a key parameter to ensure the stable operation of the power system. Currently, the stress in the cold shrink tube during the installation of cable accessories relies on subjective methods such as visual observation and finger touch, lacking accurate and reliable detection means.

Non-destructive testing methods for residual stress in materials include X-ray diffraction [1], neutron diffraction [2], and laser interferometry. Among them, X-ray methods are limited by the measurement platform, making it difficult to perform on-site measurements of parts. Neutron diffraction has low measurement efficiency and requires a reactor or accelerator to provide a neutron source. The measurement equipment is expensive and bulky, remaining in the laboratory stage, unable to achieve large-scale measurements in manufacturing environments. Acoustic detection, as an important non-invasive detection method, has advantages such as good directivity, high resolution, and strong real-time performance, making certain progress in material stress detection.

Critically refracted longitudinal waves (LCR waves) are a type of ultrasonic wave. When ultrasonic longitudinal waves are obliquely incident on solid materials with higher wave speeds, the waves refract. When the incident angle is the first critical refraction angle, the longitudinal waves propagating on the material surface are LCR waves. Compared to other ultrasonic wave modes, LCR wave speeds are more sensitive to material stress changes. The LCR wave measurement method was initially proposed by Srinivasan et al. [3] and has become the most commonly used ultrasonic method for residual stress measurement [4,5,6,7,8,9]. Since LCR waves propagate along the material surface, they are mainly used for measuring near-surface residual stress in parts. In unidirectional residual stress fields, Jin et al. [10] measured the sensitivity of LCR wave speeds to stress along different directions. Javadi et al. [11] experimentally studied the measurement depth of LCR waves using the notch method, showing that the measurement depth decreases with increasing ultrasonic frequency. Wang et al. [12] proposed an improved ultrasonic method for plane stress measurement using LCR waves and applied a cruciform specimen method combined with a digital image correlation method to confirm the validity of the LCR method. Notably, LCR waves reflect stress changes based on an average value over the acoustic propagation channel, making it difficult to detect stress changes in different regions of the material. Additionally, the application of LCR waves is mainly limited to rigid materials with low acoustic impedance, posing significant limitations in measuring the stress in the cold shrink tube of cable accessories.

When the wavelength of an ultrasonic wave is comparable to the thickness, diameter, and other characteristic dimensions of the part to be measured, the wave undergoes constant reflection, refraction, interference, and mode conversion at the medium boundaries, forming ultrasonic guided waves, such as Lamb waves. Lamb waves exhibit low energy attenuation and long propagation distances, enabling large-area, high-efficiency stress measurement. Safikhanlu et al. [13] measured the stress in 1050 aluminum samples with a thickness of 0.5 mm, showing that the changes in flight time of S0 and A0 Lamb waves are 3.75 and 1.91 times that of LCR waves, respectively, making them more sensitive to stress measurement. Lim et al. [14] proposed a dynamic and static load online stress monitoring technology for metal plate structures based on Lamb wave measurement and convolutional neural networks (CNNs). However, Lamb waves exhibit complex dispersion characteristics during propagation, posing numerous problems and challenges in material stress inversion. 

Pulse-echo ultrasonic longitudinal wave is an alternative method for stress evaluation. This technology is based on the acoustoelastic effect, where the ultrasonic wave propagation speed is linked to the magnitude of the stresses. Vangi [15] utilized longitudinal waves in reflection that propagate in a direction perpendicular to the surface of the workpiece and achieved stress measurement in different experimental configurations. The combination of ultrasonic transverse and longitudinal wave testing methods also has new applications in plane stress measurement [16], but methods based on the attenuation of ultrasonic longitudinal waves for material application measurements are rarely reported.

This paper explores a method for measuring the stress in the cold shrink tube of high-voltage cable accessories based on the attenuation of ultrasonic longitudinal waves. By measuring the transmission and attenuation characteristics of ultrasonic longitudinal waves in multi-layer elastic insulation materials and combining it with theoretical calculations of radial stress in the cold shrink tube, effective measurement of the stress in high-voltage cable accessories can be achieved, providing key technical support for improving the installation process of high-voltage cable accessories.

## 2. Ultrasonic Testing System

**Figure 1:**
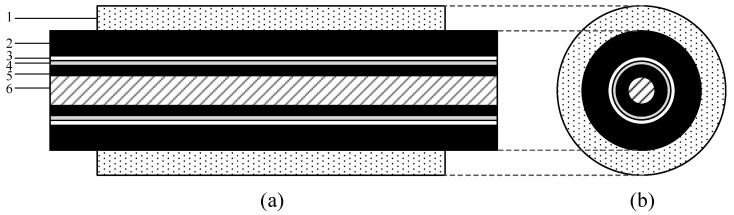
Schematic diagram of multi-layer structure of cable accessories: (**a**) front view and (**b**) side view (1: cold shrink tube; 2: PVC outer sheath; 3: wrapping tape; 4: copper armor; 5: XLPE insulation layer; and 6: aluminum core).

Figure 1 shows the structure of a 35 kV aluminum core cable and a schematic diagram of the cable accessory. The 35 kV aluminum core cable consists of multiple layers: aluminum core, cross-linked polyethylene (XLPE) insulation layer, copper armor, wrapping tape, and polyvinyl chloride (PVC) outer sheath. The cold shrink tube of the cable accessory is made of liquid silicone rubber, mainly composed of vinyl group-containing polydimethylsiloxane, a platinum catalyst, and an inhibitor.

**Figure 2:**
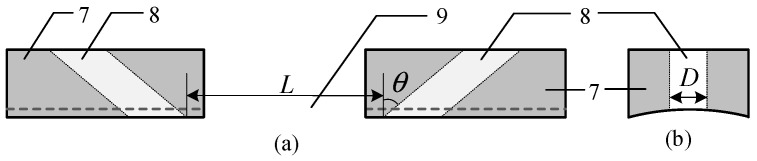
Schematic diagram of ultrasonic probe fixing bracket: (**a**) front view and (**b**) side view (7: bracket; 8: through hole for placing ultrasonic probe; and 9: air gap).

Figure 2 is a schematic diagram of the ultrasonic probe fixing bracket, which is made of plexiglass and used to fix the ultrasonic probe and the object to be measured. Thus, a certain angle and distance between them can be ensured for the emission and reception of ultrasonic signals. The emission probe and reception prob used in the experiment are selected from ULSO Corporation (type: UC3P6S) and their wear face is impedance matched to water, which significantly enhances the energy transfer between the probe and the coupling medium, thereby reducing reflection losses and improving measurement accuracy. The diameter *D* is 8 mm, with a distance *L* of 30 mm between them. The emission and reception probes form an angle *θ* with the vertical direction. This design of size and spacing ensures effective coverage of ultrasonic wave propagation within the cold shrink tube, thereby increasing the system’s adaptability.

**Figure 3:**
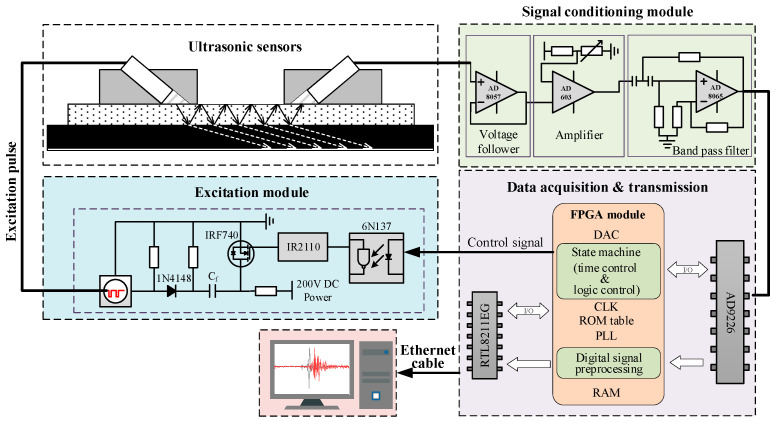
Schematic diagram of the measurement system for cold shrink tube stress based on ultrasonic longitudinal wave attenuation.

**Figure 4:**
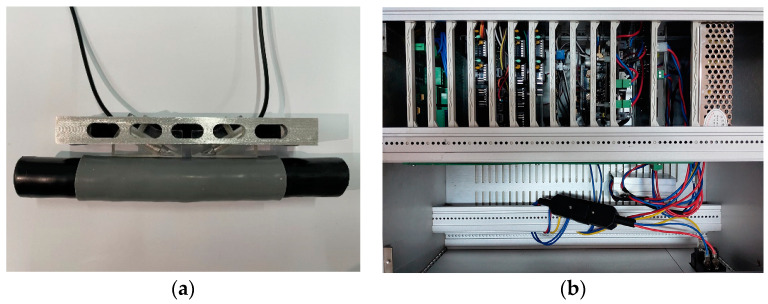
Measurement system for the cold shrink tube stress: (**a**) ultrasonic probes and (**b**) FPGA module and data acquisition and transmission module.

Figure 3 is a schematic diagram of the measurement system for cold shrink tube stress based on ultrasonic longitudinal wave attenuation. Ultrasonic longitudinal waves propagate in the cold shrink tube, accompanied by multiple reflections and refractions at layered interfaces, resulting in ultrasonic wave energy attenuation. This paper attempts to measure the stress in the cold shrink tube using the attenuation of received ultrasonic waves. The testing system mainly includes four modules: FPGA module, excitation module, signal conditioning module, data acquisition and transmission module. The FPGA module (Altera Cyclone IV EP4CE6E22C8N) generates a control signal (square signal) with a repetition frequency of 50 Hz. The excitation module produces high-voltage pulse signals under the control signal to drive the ultrasonic emission probe to emit ultrasonic waves at a frequency of 3 MHz. After propagating through the cold shrink tube, the ultrasonic waves are converted into electrical signals by the ultrasonic reception probe. These signals are amplified and filtered by the conditioning module. The output signal of the conditioning module was converted into a digital signal by 12-bit high-speed ADC (AD9226) operating at 40 MHz. The digitized signal was then fed into the FPGA chip for storage, and subsequently uploaded to the host computer via an Ethernet connection. Details about the measurement systems can be found in [17,18]. See Figure 4.

## 3. Theoretical Calculation of Multi-Interface Ultrasonic Longitudinal Wave Attenuation

**Figure 5:**
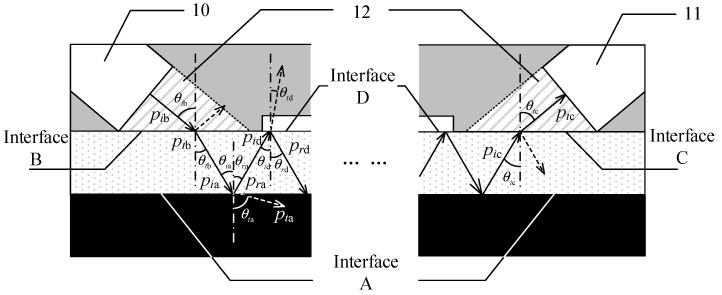
Schematic diagram of ultrasonic longitudinal wave reflection and refraction at interfaces (10: ultrasonic emission probe; 11: ultrasonic reception probe; 12: coupling agent).

Figure 5 shows the schematic diagram of ultrasonic longitudinal wave interface reflection and refraction. For the cold shrink tube–PVC interface (interface A), according to the theory of reflection and refraction of oblique incident ultrasonic waves, a part of the sound wave will reflect back into the cold shrink tube at a certain angle, while another part will penetrate into the PVC medium. According to Snell’s law of sound wave reflection and refraction [19],

(1) $$\theta_{ia}=\theta_{ra}$$

(2) $$\frac{\sin\theta_{ia}}{\sin\theta_{ta}}=\frac{c_{1}}{c_{2}}$$

where $\theta_{ia}$, $\theta_{ra}$, and $\theta_{ta}$ are the angles of incidence, reflection, and refraction of the sound wave at the cold shrink tube–PVC interface, respectively. $c_{1}$ and $c_{2}$ are the sound speeds in the cold shrink tube and PVC medium, respectively.

At the same time, the ratios of the reflected wave sound pressure $p_{ra}$ to the incident wave sound pressure and the transmitted wave sound pressure $p_{ta}$ to the incident wave sound pressure at this interface are as follows:

(3) $$r_{pa}=\frac{p_{ra}}{p_{ia}}=\frac{\rho_{2}c_{2}\cos\theta_{ia}-\rho_{1}c_{1}\cos\theta_{ta}}{\rho_{2}c_{2}\cos\theta_{ia}+\rho_{1}c_{1}\cos\theta_{ta}}$$

(4) $$t_{pa}=\frac{p_{ta}}{p_{ia}}=\frac{2\rho_{2}c_{2}\cos\theta_{ia}}{\rho_{2}c_{2}\cos\theta_{ia}+\rho_{1}c_{1}\cos\theta_{ta}}$$

where $\rho_{1}$ and $\rho_{2}$ denote the densities of the cold shrink tube and polyvinyl chloride, respectively.

Similarly, based on Snell’s law and the oblique incidence theory of ultrasonic longitudinal waves, the refraction angles $\theta_{tb}$, $\theta_{tc}$, and $\theta_{td}$ at the liquid coupling agent–cold shrink tube interface, cold shrink tube–liquid coupling agent interface, and cold shrink tube–air interface, respectively, can be obtained. The reflection angle $\theta_{rd}$ at the interface D and the ratios of the transmitted wave sound pressure to the incident wave sound pressure $t_{pb}$, $t_{pc}$, and the ratio of the reflected wave sound pressure to the incident wave sound pressure, $r_{pd}$, at the interface D are as follows:

(5) $$\frac{\sin\theta_{ib}}{\sin\theta_{tb}}=\frac{c_{L}}{c_{1}}$$

(6) $$\frac{\sin\theta_{ic}}{\sin\theta_{tc}}=\frac{c_{1}}{c_{L}}$$

(7) $$\frac{\sin\theta_{id}}{\sin\theta_{td}}=\frac{c_{1}}{c_{g}}$$

(8) $$\theta_{id}=\theta_{rd}$$

(9) $$t_{pb}=\frac{p_{tb}}{p_{ib}}=\frac{2\rho_{1}c_{1}\cos\theta_{ib}}{\rho_{1}c_{1}\cos\theta_{ib}+\rho_{L}c_{L}\cos\theta_{tb}}$$

(10) $$t_{pc}=\frac{p_{tc}}{p_{ic}}=\frac{2\rho_{L}c_{L}\cos\theta_{ic}}{\rho_{L}c_{L}\cos\theta_{ic}+\rho_{1}c_{1}\cos\theta_{tc}}$$

(11) $$r_{pd}=\frac{p_{rd}}{p_{id}}=\frac{\rho_{g}c_{g}\cos\theta_{id}-\rho_{1}c_{1}\cos\theta_{td}}{\rho_{g}c_{g}\cos\theta_{id}+\rho_{1}c_{1}\cos\theta_{td}}$$

where $\rho_{L}$ and $\rho_{g}$ are the densities of the liquid coupling agent and air, respectively, and $c_{L}$ and $c_{g}$ are the sound speeds in the liquid coupling agent and air, respectively. The angles $\theta_{ib}$, $\theta_{ic}$, and $\theta_{id}$ are the incident angles of sound waves at the liquid coupling agent–cold shrink tube interface, cold shrink tube–liquid coupling agent interface, and cold shrink tube–air interface, respectively. Meanwhile, $p_{tb}$ and $p_{ib}$ as well as $p_{tc}$ and $p_{ic}$ represent the transmitted wave sound pressures and incident wave sound pressures at interfaces B and C, and $p_{rd}$ and $p_{id}$ represent the reflected wave sound pressures and incident wave sound pressures at the interface D.

The incident angle $\theta_{ib}$ at interface B is the angle between the emission probe and the vertical direction θ. The incident angles $\theta_{ia}$, $\theta_{ic}$, and $\theta_{id}$ at interfaces A, C, and D are all equal to the refraction angle $\theta_{tb}$ at the liquid coupling agent–cold shrink tube interface.

The number of reflections, *N*, of the ultrasonic longitudinal wave in the cold shrink tube changes with the thickness of the cold shrink tube, δ, and can be expressed as follows:

(12) $$N=\frac{L}{\delta \cdot \tan\theta_{tb}}$$

During the reflection process, the number of reflections of the ultrasonic longitudinal wave at interface A is *N*/2 and at interface D is *N*/2−1. The number of reflections, *N*, of the ultrasonic longitudinal wave is negatively correlated with the sound pressure p at the ultrasonic reception probe, meaning the larger the number of reflections, the smaller the signal amplitude received by the probe.

The length of the propagation path of the ultrasonic wave in the cold shrink tube is $L/\sin\theta_{tb}$. Given that the attenuation coefficient of ultrasound in the cold shrink tube is $\alpha_{1}$, the attenuation $e^{-\alpha_{1}\cdot \frac{L}{\sin\theta_{tb}}}$ of the ultrasonic wave along the propagation path can be obtained.

The incident wave sound pressure at interface B, $p_{ib}$, is the emission sound pressure $p_{i}$ of the ultrasonic probe, and the transmitted wave sound pressure at interface C, $p_{tc}$, is the reception sound pressure p of the ultrasonic probe. Therefore, the relationship between them can be summarized as follows:

(13) $$p=t_{pb}\cdot t_{pc}\cdot {\left(r_{pd}\right)}^{\frac{N}{2}-1}\cdot {\left(r_{pa}\right)}^{\frac{N}{2}}\cdot e^{-\alpha_{1}\cdot \frac{L}{\sin\theta_{tb}}}p_{i}$$

Because there is a linear relationship between the sound pressure of the ultrasonic probe and the signal amplitude of the ultrasonic probe, the relationship between the emission signal amplitude $A_{i}$ of the ultrasonic probe and the reception signal amplitude A can be established as follows:

(14) $$A=t_{pb}\cdot t_{pc}\cdot {\left(r_{pd}\right)}^{\frac{L}{2\delta \cdot \tan\theta_{tb}}-1}{\left(r_{pa}\right)}^{\frac{L}{2\delta \cdot \tan\theta_{tb}}}\cdot e^{-\alpha_{1}\cdot \frac{L}{\sin\theta_{tb}}}A_{i}$$

For the same specifications of cold shrink tube samples in a fully relaxed state, with a cold shrink tube thickness of $\delta_{0}$, and with the cold shrink tube being empty, the ultrasonic wave during the reflection process only collides with the cold shrink tube–air interface. At this time, the transmitted wave sound pressure at the C interface, $p_{tc}$, is the reception sound pressure $p_{0}$, and the relationship between $p_{0}$ and $p_{i}$ can be expressed as follows:

(15) $$p_{0}=t_{pb}\cdot t_{pc}\cdot {\left(r_{pd}\right)}^{\frac{N}{2}-1}\cdot e^{-\alpha_{1}\cdot \frac{L}{\sin\theta_{tb}}}p_{i}$$

Therefore, the relationship between the emission signal amplitude $A_{i}$ and reception signal amplitude $A_{0}$ of the ultrasonic probe under the fully relaxed state of the cold shrink tube can be obtained as follows:

(16) $$A_{0}=t_{pb}\cdot t_{pc}\cdot {\left(r_{pd}\right)}^{\frac{L}{\delta_{0}\cdot \tan\theta_{tb}}-1}\cdot e^{-\alpha_{1}\cdot \frac{L}{\sin\theta_{tb}}}A_{i}$$

From Equations (14) and (16), the relative amplitude $A/A_{0}$ can be derived as follows:

(17) $$A/A_{0}=\frac{{\left(r_{pa}\right)}^{\frac{L}{2\delta \cdot \tan\theta_{tb}}}{\left(r_{pd}\right)}^{\frac{L}{2\delta \cdot \tan\theta_{tb}}-1}}{{\left(r_{pd}\right)}^{\frac{L}{\delta_{0}\cdot \tan\theta_{tb}}-1}}$$

This relative amplitude $A/A_{0}$ can eliminate the interference of reflection attenuation at interfaces B and C and the path attenuation of the ultrasonic wave. The smaller the value of $A/A_{0}$, the greater the attenuation degree of the ultrasonic wave.

According to Equation (17), the theoretical relationship between the relative thickness $\delta /\delta_{0}$ of the cold shrink tube and the relative amplitude $A/A_{0}$ can be obtained as follows:

(18) $$\delta /\delta_{0}=\frac{L\cdot \left(\ln(r_{pa})+\ln(r_{pd})\right)}{2\cdot \tan\theta_{tb}\cdot \delta_{0}\cdot \ln(A/A_{0})+L\cdot \ln(r_{pd})}$$

where $\delta_{0}$ is the thickness of the cold shrink tube in the fully relaxed state.

## 4. Experimental Calibration of Ultrasonic Longitudinal Wave Attenuation in Multiple Interfaces

**Figure 6:**
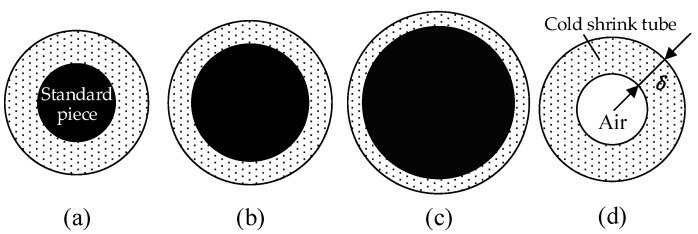
Schematic diagram of standard pieces for cold shrink tube thickness measurement: (**a**) δ = 2.2 mm; (**b**) δ = 1.6 mm; (**c**) δ = 1 mm; and (**d**) δ = 2.2 mm, air calibration.

Figure 6 shows diagrams of cold shrink tubes and PVC standard pieces under four typical conditions. Using cold shrink tubes of the same specification to wrap the PVC standard pieces, the thickness δ of the cold shrink tubes decreases as the diameter $d_{\mathrm{PVC}}$ of the PVC standard piece increases, as shown in Figure 6a–c. Additionally, a sample made with a cold shrink tube of the same specification is shown in a completely relaxed state with air inside the tube, as illustrated in Figure 6d.

**Figure 7:**
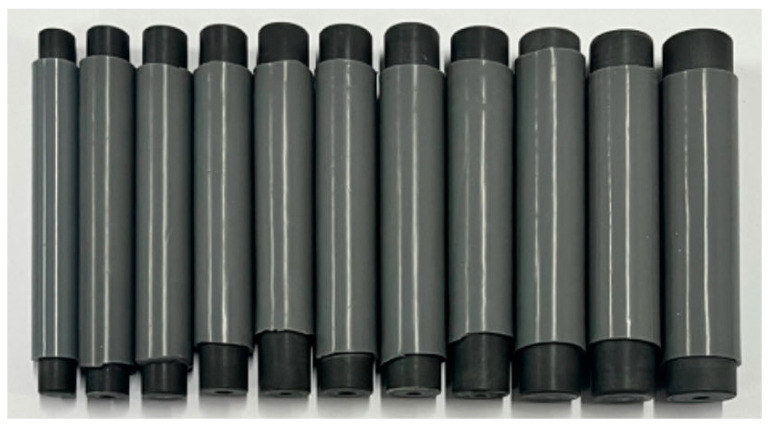
Cold shrink tube wrapping PVC standard pieces (showing only 11 pieces).

**Figure 8:**
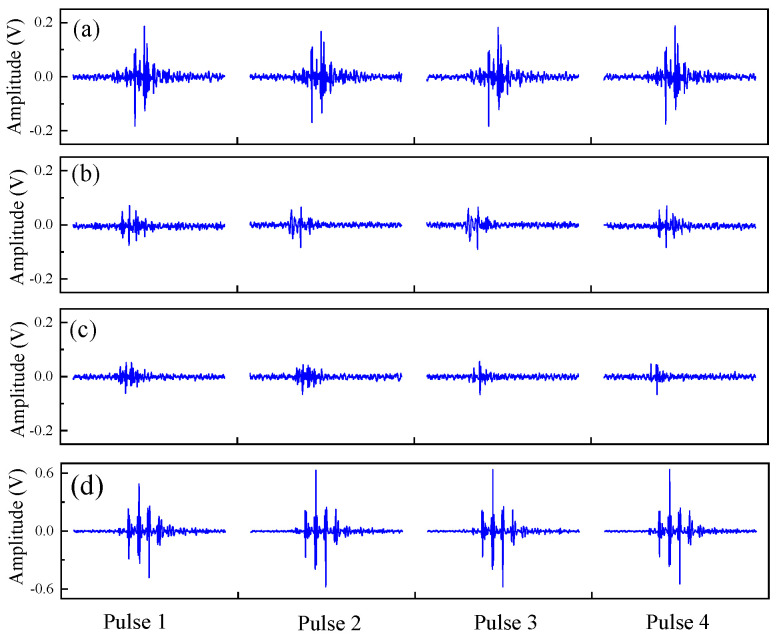
Ultrasonic pulse sequences under different cold shrink tube thicknesses: (**a**) δ = 2.2 mm; (**b**) δ = 1.6 mm; (**c**) δ = 1 mm; and (**d**) δ = 2.2 mm, air calibration.

The diameter of the PVC standard pieces used in the calibration experiment ranges from 14 mm to 30 mm, with increments of 1 mm, resulting in a total of 17 experimental samples, as shown in Figure 7. Three sampling positions were randomly selected for each standard piece, and three measurements were performed at each sampling position, resulting in a total of 153 groups of ultrasonic response signals, each containing 500 ultrasonic pulse sequences. At the same time, pulse sequences of the cold shrink tube in a fully relaxed state were measured. Figure 8 shows the ultrasonic pulse sequences under four typical conditions. It can be seen from Figure 8a–c that, as the thickness of the cold shrink tube decreases, the amplitude of the ultrasonic pulse gradually decreases, indicating a monotonic relationship between the two. From Figure 8d, it can be seen that, when the cold shrink tube is empty, its response signal amplitude is significantly greater than that of a tube of the same thickness wrapped with PVC.

The maximum values of the 500 ultrasonic pulse sequences in each group of ultrasonic response signals are extracted and averaged, denoted as A. At the same time, the maximum value of the ultrasonic pulse sequence is extracted and averaged when the cold shrink tube is in a relaxed state and empty, denoted as $A_{0}$. Thus, the relative amplitude $A/A_{0}$ is obtained.

**Figure 9:**
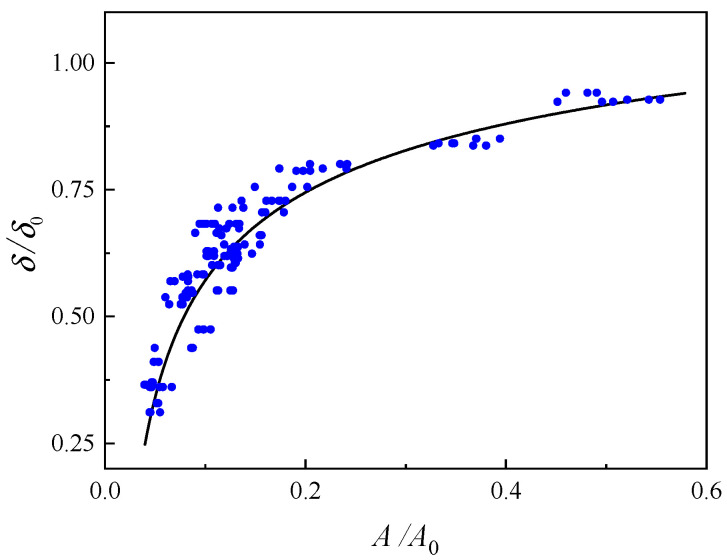
Relationship between ultrasonic wave attenuation and the relative thickness of the cold shrink tube.

The relative amplitude $A/A_{0}$ measured in the calibration experiment is shown in Figure 9, where each point corresponds to a group of ultrasonic response signals. By referring to the function model of Equation (19), the relationship between the relative thickness $\delta /\delta_{0}$ of the cold shrink tube and the relative amplitude $A/A_{0}$ can be determined as follows:

(19) $$\delta /\delta_{0}=\frac{-6.86}{\ln(A/A_{0})+7.19}+1.972$$

## 5. Results of Cold Shrink Tube Stress Measurement

### 5.1. Theoretical Relationship between Cold Shrink Tube Thickness and Radial Stress

**Figure 10:**
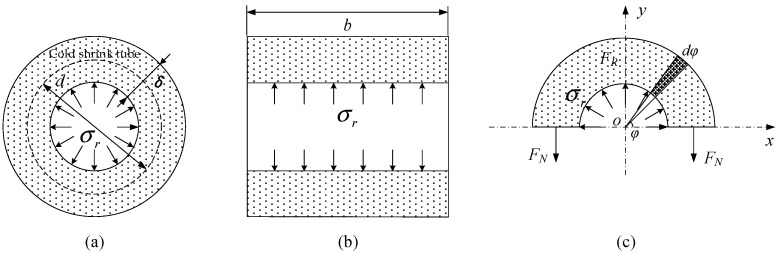
Radial stress diagram of the cold shrink tube: (**a**) the cross-sectional view of the cold shrink tube; (**b**) the axial cross-sectional view of the cold shrink tube; and (**c**) the cross-sectional view of the upper half of the cold shrink tube.

The radial stress, $\sigma_{r}$, applied by the cable to the cold shrink tube during shrinkage is shown in Figure 10. The initial thickness of the cold shrink tube in a relaxed state is $\delta_{0}$, the initial diameter of the cross-sectional circle is $d_{0}$, and the initial longitudinal length is $b_{0}$. When it shrinks and is fixed on the cable, it expands uniformly under the internal force, with thickness δ, diameter d, and longitudinal length b. For any cross-section, the calculation formulas for radial strain $\varepsilon_{r}$, hoop strain $\varepsilon_{\theta }$, and longitudinal strain $\varepsilon_{z}$ are as follows:

(20) $$\left\{ \begin{array}{l} \varepsilon_{r}=\frac{\delta -\delta_{0}}{\delta_{0}}\\ \varepsilon_{z}=\frac{b-b_{0}}{b_{0}}\\ \varepsilon_{\theta }=\frac{d-d_{0}}{d_{0}} \end{array}\right.$$

According to the generalized Hooke’s law in cylindrical coordinates [20], the relationship between strains and stresses can be obtained as follows:

(21) $$\left\{ \begin{array}{l} \varepsilon_{r}=\frac{1}{E}(\sigma_{r}-\nu \sigma_{\theta }-\nu \sigma_{z})\\ \varepsilon_{\theta }=\frac{1}{E}(\sigma_{\theta }-\nu \sigma_{r}-\nu \sigma_{z})\\ \varepsilon_{z}=\frac{1}{E}(\sigma_{z}-\nu \sigma_{r}-\nu \sigma_{\theta }) \end{array}\right.$$

Here, E is the Young’s modulus, ν is Poisson’s ratio, and $\sigma_{\theta }$ and $\sigma_{z}$ are the hoop stress and longitudinal stress, respectively. By combining Equations (20) and (21), the calculation formula for hoop stress $\sigma_{\theta }$ can be derived as follows:

(22) $$\sigma_{\theta }=\frac{E\nu \varepsilon_{r}+(1-\nu )E\varepsilon_{\theta }+E\nu \varepsilon_{Z}}{-2\nu^{2}-\nu +1}$$

In Figure 10c, the upper half of the cold shrink tube is analyzed after being longitudinally cut. Taking a microelement dφ of the cold shrink tube rotated by an angle around the center O as the study object, each segment experiences radial force due to the shrinkage of the cold shrink tube. Integrating the component of this force in the *y*-axis direction from 0 to π, the resultant radial force is $F_{R}$. According to the force balance relationship in the y-direction,

(23) $$F_{R}=2F_{N}$$

where *F_N_* is the circumferential force, calculated by the formula $F_{N}=\sigma_{\theta }\cdot \left(b\cdot \delta \right)$. Thus, $F_{R}$ can be expressed as follows:

(24) $$F_{R}=2b\cdot \delta \frac{E\nu \varepsilon_{r}+(1-\nu )E\varepsilon_{\theta }+E\nu \varepsilon_{Z}}{-2\nu^{2}-\nu +1}$$

Since the constraint force caused by the shrinkage of the cold shrink tube does not cause longitudinal strain, $\varepsilon_{z}$ can be ignored. Moreover, based on the characteristic that the volume of the cold shrink tube remains unchanged before and after shrinkage, we have $d/d_{0}=\delta_{0}/\delta$. Therefore, the relationship between the resultant radial force $F_{R}$ in the y-direction and the thickness δ can be obtained as follows:

(25) $$F_{R}=2\frac{b\cdot E}{\delta_{0}}\cdot \frac{\nu \delta^{2}-\delta_{0}\cdot \delta +(1-\nu )\delta_{0}^{2}}{-2\nu^{2}-\nu +1}$$

**Figure 11:**
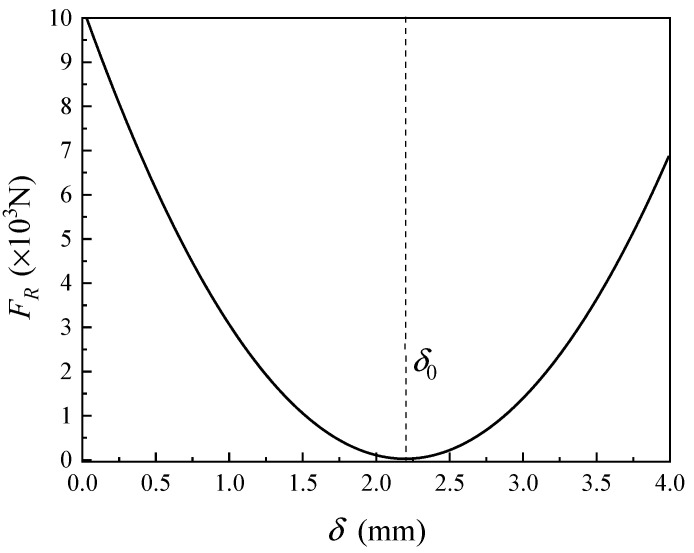
Relationship between the resultant radial force in the y-direction and the thickness of the cold shrink tube.

Given that the Young’s modulus E of the cold shrink tube is 7 MPa, the Poisson’s ratio *ν* is 0.4995, and the initial thickness $\delta_{0}$ of the cold shrink tube used in this experiment is 2.2 mm, for a cold shrink tube segment with a longitudinal length b=1 mm, the relationship between the resultant radial force $F_{R}$ in the y-direction and the thickness δ can be simplified as shown in Equation (26). The function graph is illustrated in Figure 11.

(26) $$F_{R}=2.12\times 10^{9}\cdot \delta^{2}-9.34\times 10^{6}\cdot \delta +1.03\times 10^{4}$$

Continuing with the micro-segment of the cold shrink tube, which rotates around the center *O* in Figure 10c by an angle dφ, as the object of study, the expansion force acting on it is a force directed away from the center along the radial direction, denoted as $\sigma_{r}\cdot \left[b\cdot \left(\frac{d}{2}\right)\cdot d\varphi \right]$. The component of this force in the *y*-axis direction is $\sigma_{r}\cdot \left[b\cdot \left(\frac{d}{2}\right)\cdot d\varphi \right]\cdot \sin\varphi$. By integrating this force from φ=0 to φ=π, the relationship between the resultant radial force $F_{R}$ in the y-direction and the radial stress $\sigma_{r}$ can be obtained [21] as follows:

(27) $$\int_{0}^{\pi }\sigma_{r}\cdot b\cdot \left(\frac{d}{2}\right)\cdot \sin\varphi \cdot d\varphi =\sigma_{r}\cdot b\cdot d=F_{R}$$

**Figure 12:**
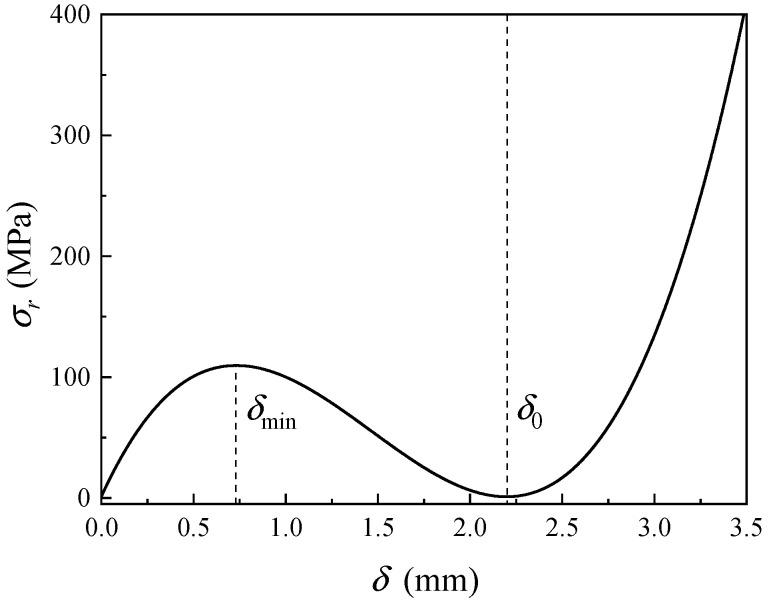
Relationship between radial stress and thickness of cold shrink tube.

Consequently, the relationship between the radial stress $\sigma_{r}$ and the thickness δ can be determined, as expressed in Equation (28). The corresponding function graph is depicted in Figure 12.

(28) $$\sigma_{r}=2\frac{E}{d_{0}\cdot \delta_{0}^{2}}\cdot \frac{\nu \delta^{3}-\delta_{0}\cdot \delta^{2}+(1-\nu )\delta_{0}^{2}\cdot \delta }{-2\nu^{2}-\nu +1}$$

It can be observed that the effective operating range of the cold shrink tube is between the minimum thickness $\delta_{\min}$ and the initial thickness $\delta_{0}$. Due to its material properties, the cold shrink tube cannot be further thickened beyond its initial thickness. When the thickness decreases below the minimum value, a series of issues may arise. First, the reduced thickness of the cold shrink tube, combined with the relatively large size of the enclosed cable and its accessories, results in decreased force per unit area. This reduction in force compromises the ability of the tube to provide a tight and reliable seal, thereby affecting its performance. Additionally, a thinner cold shrink tube increases the risk of electrical breakdown, as it may not offer sufficient insulation for high-voltage transmission. Moreover, the reduced thickness also diminishes the mechanical strength of the tube, potentially weakening its resistance to environmental factors such as moisture ingress, UV radiation, and mechanical wear. Over time, these factors could accelerate the degradation of the cold shrink tube, further impairing its performance.

### 5.2. Cold Shrink Tube Stress Measurement

By substituting the relationship between the relative amplitude of the ultrasonic pulse and the relative thickness of the cold shrink tube, i.e., Equation (19), into the formula for the radial stress $\sigma_{r}$ and the thickness δ, i.e., Equation (28), the relationship between the relative amplitude $A/A_{0}$ of the ultrasonic pulse and the radial stress $\sigma_{r}$ can be obtained.

**Figure 13:**
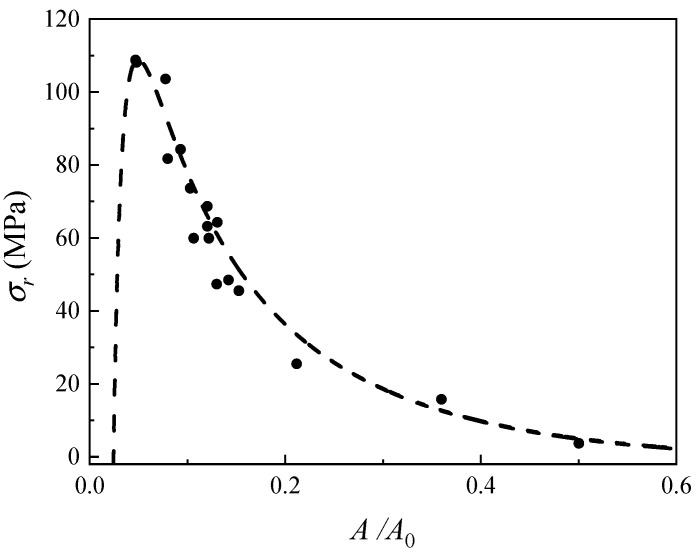
Measurement results of cold shrink tube stress.

Figure 13 shows the comparison between the measured and theoretical values of the radial stress $\sigma_{r}$. The black dots in the figure correspond to the measurement results of 17 groups of experimental samples. The horizontal axis represents the average value of the relative amplitude $A/A_{0}$ of the ultrasonic pulses of the 17 groups of experimental samples. The dashed line represents the theoretical value of the radial stress $A/A_{0}$ calculated based on the average thickness of each group of experimental samples. It can be seen that the measured stress values of the cold shrink tube agree well with the theoretical values, and the measurement system shows high sensitivity when the relative amplitude $A/A_{0}$ of the ultrasonic pulse ranges from 0.05 to 0.5.

## 6. Conclusions

The stress of the cold shrink tube in high-voltage cable accessories is one of the key parameters to ensure the stable operation of the power system. This paper explores a method for measuring the stress of high-voltage cable accessories’ cold shrink tube based on ultrasonic longitudinal wave attenuation. Firstly, a pulse ultrasonic longitudinal wave testing system based on FPGA was designed, where the ultrasonic sensor adopts a single-send, single-receive mode, with an ultrasonic frequency and pulse repetition frequency of 3 MHz and 50 Hz, respectively. The system was optimized to account for the multi-layer structural characteristics of high-voltage cable accessories, ensuring effective wave propagation within the cold shrink tube.

Ultrasonic longitudinal waves attenuate due to reflection and refraction at multi-layer interfaces. This paper divides the attenuation of ultrasonic longitudinal waves into five parts: liquid couplant–cold shrink tube interface attenuation, cold shrink tube–PVC interface attenuation, cold shrink tube–air interface attenuation, cold shrink tube internal propagation attenuation, and cold shrink tube–liquid couplant interface attenuation. The transmission and attenuation characteristics of ultrasonic longitudinal waves in multi-layer elastic media were theoretically studied, and it was found that there is an exponential relationship between ultrasonic longitudinal wave attenuation and the thickness of the cold shrink tube. Through experiments, a measurement model for the thickness of the cold shrink tube was obtained. By establishing a theoretical model of the radial stress of the cold shrink tube and using the thickness of the cold shrink tube as an intermediate variable, an effective measurement of the stress of the cold shrink tube was achieved. 

This method not only provides a more accurate and reliable means of stress monitoring in cold shrink tubes but also demonstrates the significant potential of multi-layer elastic media. Future research could focus on expanding its applicability to different materials and structures, such as composite laminate. The advancements could enhance the versatility and effectiveness of ultrasonic longitudinal wave attenuation in stress measurement.

## Data Availability

Data available upon request from the authors.

## References

[1] Sasaki T. (2014). New Generation X-Ray Stress Measurement Using Debye Ring Image Data by Two-Dimensional Detection. Mater. Sci. Forum.

[2] Mo F., Sun G., Li J., Zhang C., Wang H., Chen Y., Liu Z., Yang Z., Li H., Yang Z. (2018). Recent Progress of Residual Stress Distribution and Structural Evolution in Materials and Components by Neutron Diffraction Measurement at RSND. Quantum Beam Sci..

[3] Srinivasan M., Chundu S., Bray D., Alagarsamy A. (1992). Ultrasonic Technique for Residual Stress Measurement in Ductile Iron Continuous Cast Round Bars. J. Test. Eval..

[4] Bray D.E., Tang W. (2001). Subsurface stress evaluation in steel plates and bars using the LCR ultrasonic wave. Nucl. Eng. Des..

[5] Bray D.E., Pathak N., Srinivasan M.N. (1996). Residual Stress Distributions in the Rim of a Steam Turbine Disk Using the LCR Ultrasonic Technique. Mater. Sci. Forum.

[6] Liu H., Li Y., Li T., Zhang X., Liu Y., Liu K., Wang Y. (2018). Influence factors analysis and accuracy improvement for stress measurement using ultrasonic longitudinal critically refracted (LCR) wave. Appl. Acoust..

[7] He J., Li Z., Teng J., Li M., Wang Y. (2018). Absolute stress field measurement in structural steel members using the Lcr wave method. Measurement.

[8] Mohammadi M., Fesharaki J.J. (2019). Determination of acoustoelastic/acoustoplastic constants to measure stress in elastic/plastic limits by using LCR wave. NDT E Int..

[9] Wang W., Zhang Y., Zhou Y., Meng S., Chen D. (2019). Plane stress measurement of orthotropic materials using critically refracted longitudinal waves. Ultrasonics.

[10] Jin C., Lu C., Shi Y.W., Liang J., Wang X. (2015). Study on relationship between critically refracted longitudinal wave and internal stress in pre-stretched aluminium alloy plate. Mater. Res. Innov..

[11] Javadi Y., Hloch S. (2013). Employing the LCR Waves to Measure Longitudinal Residual Stresses in Different Depths of a Stainless Steel Welded Plate. Adv. Mater. Sci. Eng..

[12] Wang W., Xu C., Zhang Y., Zhou Y., Meng S., Deng Y. (2018). An improved ultrasonic method for plane stress measurement using critically refracted longitudinal waves. NDT E Int..

[13] Safikhanlu H., Alimirzaei S., Ahmadi Najafabadi M. (2020). Behavior of Critically Refracted Longitudinal and Lamb Waves with Stress Variations in Al 1050 Sample. Modares Mech. Eng..

[14] Lim H.J., Sohn H. (2020). Online Stress Monitoring Technique Based on Lamb-wave Measurements and a Convolutional Neural Network Under Static and Dynamic Loadings. Exp. Mech..

[15] Vangi D. (2001). Stress evaluation by pulse-echo ultrasonic longitudinal wave. Exp. Mech..

[16] Wang Y., Zhu X., Gong Y., Liu N., Li Z., Long Z., Teng J. (2022). Combination of transverse and longitudinal ultrasonic waves for plane stress measurement of steel plates. Appl. Acoust..

[17] Zhai L., Xia H., Xie H., Yang J. (2021). Structure detection of horizontal gas–liquid slug flow using ultrasonic transducer and conductance sensor. IEEE Trans. Instrum. Meas..

[18] Zhai L., Xu B., Xia H., Jin N. (2023). Simultaneous measurement of velocity profile and liquid film thickness in horizontal gas–liquid slug flow by using ultrasonic Doppler method. Chin. J. Chem. Eng..

[19] Slawinski M.A., Slawinski R.A., Brown R.J., Parkin J.M. (2000). A generalized form of Snell’s law in anisotropic media. Geophysics.

[20] Glass E.N., Winicour J. (1973). A geometric generalization of Hooke’s law. J. Math. Phys..

[21] Niu W., Ye W., Chen B., Huang Y. (2010). A calculating method for the inside swelling pressure of elastic circular rubber barrel according to swelling deformation. Eng. Mech..

